# Data-driven time-dependent state estimation for interfacial fluid mechanics in evaporating droplets

**DOI:** 10.1038/s41598-021-92965-8

**Published:** 2021-06-30

**Authors:** Sahar Andalib, Kunihiko Taira, H. Pirouz Kavehpour

**Affiliations:** Department of Mechanical and Aerospace Engineering, University of California, Los Angeles, CA 90095 USA

**Keywords:** Fluid dynamics, Scientific data

## Abstract

Droplet evaporation plays crucial roles in biodiagnostics, microfabrication, and inkjet printing. Experimentally studying the evolution of a sessile droplet consisting of two or more components needs sophisticated equipment to control the vast parameter space affecting the physical process. On the other hand, the non-axisymmetric nature of the problem, attributed to compositional perturbations, introduces challenges to numerical methods. In this work, droplet evaporation problem is studied from a new perspective. We analyze a sessile methanol droplet evolution through data-driven classification and regression techniques. The models are trained using experimental data of methanol droplet evolution under various environmental humidity levels and substrate temperatures. At higher humidity levels, the interfacial tension and subsequently contact angle increase due to higher water uptake into droplet. Therefore, different regimes of evolution are observed due to adsorption–absorption and possible condensation of water which turns the droplet from a single component into a binary system. In this work, machine learning and data-driven techniques are utilized to estimate the regime of droplet evaporation, the time evolution of droplet base diameter and contact angle, and level of surrounding humidity. Droplet regime is estimated by classification algorithms through point-by-point analysis of droplet profile. Decision tree demonstrates a better performance compared to Naïve Bayes (NB) classifier. Additionally, the level of surrounding humidity, as well as the time evolution of droplet base diameter and contact angle, are estimated by regression algorithms. The estimation results show promising performance for four cases of methanol droplet evolution under conditions unseen by the model, demonstrating the model’s capability to capture the complex physics underlying binary droplet evolution.

## Introduction

Wetting and spreading of liquid on a solid surface is an omnipresent phenomenon in nature and engineering technologies such as biodiagnostics, inkjet printing, microfabrication, spray cooling, and agricultural irrigation^1–13^. Droplet evaporation has gained increasing attention over the past 25 years^14–19^. The original work^20^ on the coffee-ring effect was followed by the comprehensive study^21^ to theoretically, experimentally, and numerically calculate the evaporation rate of sessile water droplets. Evaporation of a single component sessile droplet is influenced by various factors such as substrate temperature^22^, environment pressure^23^, surfactant concentration^24^, substrate thermal conductivity^25–27^, and surrounding gas^28^. When an organic fluid droplet evaporates on a solid surface, thermocapillary instabilities known as hydrothermal waves (HTWs) are created due to surface tension gradient along the interface^29^.

Although the evolution of a single component droplet is mainly understood, the physics becomes complex when there is more than one component in the droplet. Three stages were reported for evaporation of water–ethanol mixture droplets where the first stage corresponded to evaporation of a more volatile component while the last stage was responsible for evaporation of a less volatile component^30^. The humidity of the surrounding plays a crucial role in evaporation of a binary sessile droplet. Two separate studies^31,32^ observed a rise in contact angle of binary mixtures which suggested possible condensation of water on droplet. Adsorption of water in ethanol and ethanol/water mixtures is also reported by time-resolved infrared spectroscopy^33^. Three different techniques of optical visualization, infrared thermography, and acoustic high-frequency echography were employed in a comprehensive study^34^ to examine the evaporation of butanol, ethanol, water/butanol, and water/ethanol droplets. Their results showed that due to the high hygroscopic power of ethanol, the humidity of the environment had a noticeable effect on the evolution of pure ethanol droplets. Heterogeneous thermal patterns alongside the evolution of acoustic reflection coefficient proved that ethanol droplet undergoes continuous water loading. The combined influence of ambient temperature and relative humidity on early stages (i.e. pinned contact angle) of ethanol droplet was also examined^35^. In another study^36^ the water loaded onto ethanol droplet was quantified by gas injection chromatography (GIC) under controlled ambient temperature and relative humidity. The observed reduction in ethanol concentration was attributed to both ethanol evaporation as well as water intake on the drop. They concluded that at low relative humidity, the main mechanism for water intake was that of adsorption–absorption, though at high relative humidity water condensation plays a more dominant role. While the changes in relative humidity are commonly imposed environmental conditions, controlling ambient temperature to tune the effect of humidity is rather an expensive method for practical applications. The effects of relative humidity on methanol droplet evaporation was regulated by adjusting the temperature of the substrate^37,38^ which is less expensive compared to controlling ambient temperature. It was concluded that increasing substrate temperature maintains the liquid–gas interface temperature above the dew point which in turn limits the water condensation on the drop. A regime map was also proposed based on droplet evolution under various environmental conditions.

As the number of components in the droplet increases, the underlying physics becomes more complex. Recently, multi-component droplet evaporation revealed new phenomena such as spontaneous nucleation of oil microdroplets, phase transition, and multi-component diffusion^39–42^. Such intricate physics with numerous parameters in play makes experimental studies sophisticated and time-consuming while requiring advanced equipment to finely control the environmental condition. On the other hand, the highly non-axisymmetric nature of the problem due to compositional inhomogeneities brings up significant challenges to numerical models.

Machine learning methods have emerged as powerful tools for analyzing a wide range of fluid mechanics problems^43,44^ such as turbulence^45–49^, phase transition^50^, ignition^51^, vortex vibrations^52,53^, and aerodynamics disturbances^54^. Image processing and pattern recognition techniques have been employed to analyze the remaining stains after evaporation of sessile droplets. Analysis of patterns in dried drops of biological fluids revealed a lot of information for medical diagnostics^55–59^. Two different studies^60,61^ showed that the chemistry of fluid and substrate can be identified by recognition of patterns in the stains. The aforementioned studies utilized machine learning techniques to indirectly measure and detect the mechanisms inside droplets from the footprint after evaporation. Here, we introduce a data-driven approach to directly analyze the dynamics of binary droplet evaporation (induced by transfer of a second component present in the atmosphere).

In the present study, we use data-driven classification and regression algorithms to analyze the time-dependent behavior of a methanol droplet at different levels of environmental humidity and temperature of the substrate. Water uptake into droplet through adsorption–absorption and possibly condensation turns methanol droplet into a binary system. Based on the environmental condition, a droplet evolves in different regimes: evaporation-dominated, transition, or condensation-dominated. The capability of the proposed model is evaluated by estimating four different parameters, namely: regime of droplet evaporation (through classification algorithms), level of surrounding humidity (through regression algorithms), time evolution of droplet base diameter (through regression algorithms), and time evolution of droplet contact angle (through regression algorithms). First, a classification algorithm is trained to estimate the regime of droplet evaporation through analysis of diameter and contact angle evolution over time. The objective of the model is to detect the regime of droplet evolution with even a single data point at a specific time. Second, a regression algorithm is utilized to detect the humidity of the surrounding by analyzing droplet evolution. The high hygroscopic nature of methanol allows greater amount of water uptake into the droplet in humid environments. The higher content of water in droplet increases the contact angle and alters the rate of change of volume. The regression model analyzes these changes and reversely estimates the humidity. Last, given the condition of the surrounding, the continuous evolution of macroscopic parameters of droplet, i.e., diameter and contact angle, is estimated. Our method shows great potential in opening up new paths to analyze more complicated multi-component droplet evolution and interfacial fluid mechanics in general. Estimating the evolution of different parameters of droplet is a crucial step in designing high quality and high-resolution finish products in droplet-based biodiagnostics, inkjet printing, and microfabrication technologies. Our proposed method provides necessary information on evaporation of organic liquid droplets under various environmental conditions with simple and easy-to-use algorithms without the need to perform complicated simulations. We show that the regime of droplet, relative humidity of surrounding, and time evolution of diameter and contact angle can be estimated under various conditions. In real-world applications anticipating the regime of droplet is of great importance as in many instances, the occurrence of one regime or the other should be avoided. For example, a droplet sitting on a surface forever is not ideal for high resolution of printing or biosensing.

## Results

### Physics of droplet evaporation

**Figure 1:**
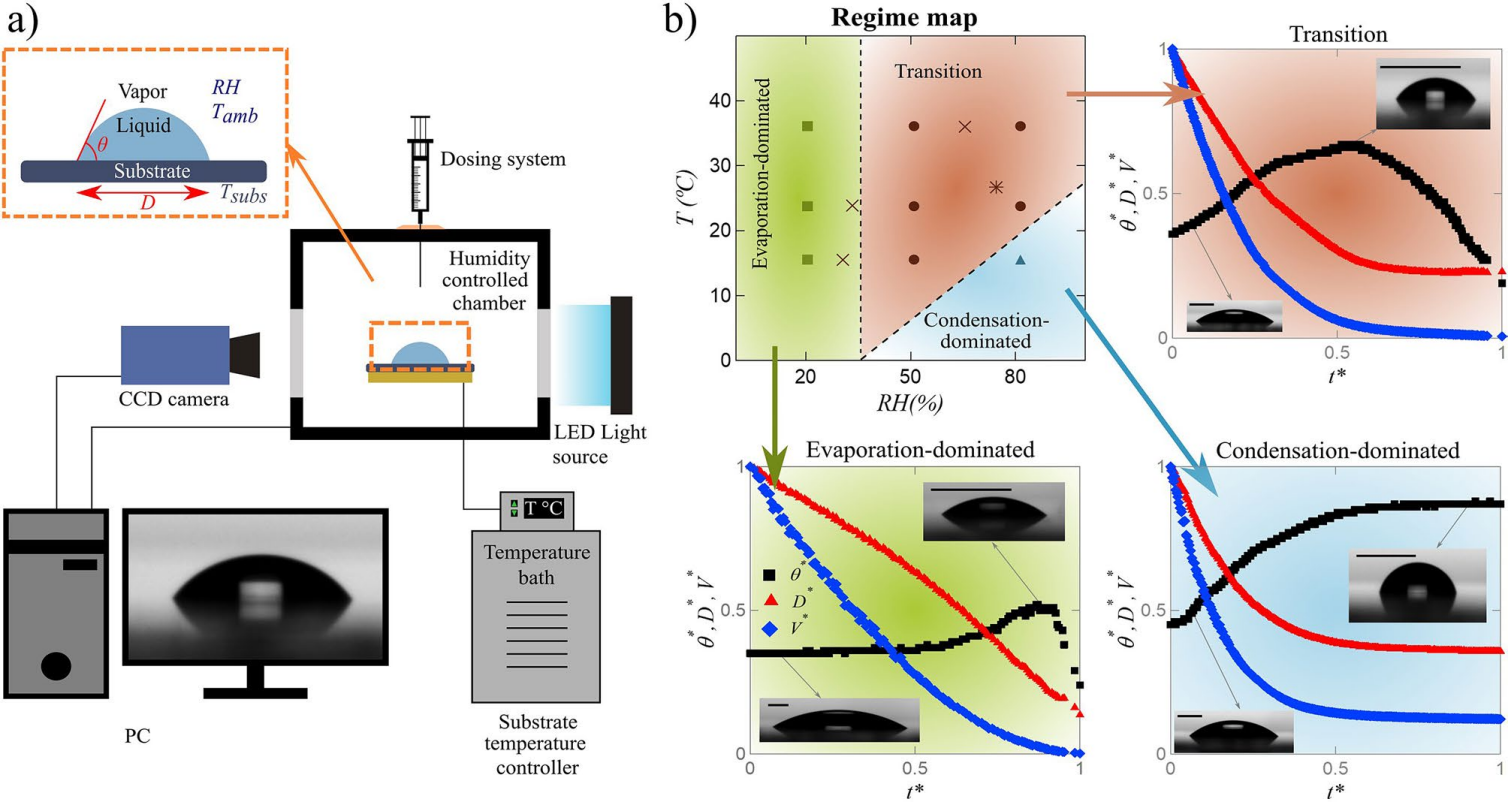
(**a**) Schematic of the experimental setup with macroscopic parameters of droplet shown in the inset; (**b**) regime map of droplet evaporation (top-left) under various relative humidity (*RH*) of surrounding and substrate temperature (*T*), evolutions of nondimensional contact angle, volume, and diameter versus time for evaporation-dominated (bottom-left), transition (top-right), and condensation-dominated (bottom-right) regimes. Each scale bar in droplet images represents a length of 1 mm. The schematic is made using free and open-source software Inkscape (Harrington^63^).

Droplet evaporation is influenced by numerous factors including liquid/substrate properties as well as environmental conditions^25–28,35,36,62^. We analyze the evolution of a sessile methanol droplet through macroscopic parameters: volume, *V*, diameter, *D*, contact angle, $\theta$, and time, *t*, under controlled relative humidity of surrounding, *RH*, and substrate temperature, *T* (see Fig. 1a, inset). The variables are nondimensionalized as: $t^* = t/t_f,\text { }V^* = V/V_0,\text { }D^* = D/D_0,\text { }\theta ^* = \theta /\theta _0,\text { }T^* = T/T_0,\text { }RH^* = RH/RH_0$, where $\theta _0 = 90^{\circ }, T_0 = 35^{\circ }C, RH_0 = 100\%$, and $t_f$, $V_0$, $D_0$, that are experimentally measured, stand for total evaporation time, initial volume, and initial diameter, respectively. The experiments are conducted in a chamber with controlled humidity and on a substrate with controlled temperature (Fig. 1a). Details of experimental procedure are given in “Methods” section. Three regimes of droplet evolution are observed under various relative humidity of the surrounding and substrate temperature (Fig. 1b, top left) namely: evaporation-dominated, transition, and condensation-dominated. Three sub figures represent the evolutions of $\theta ^*$, $V^*$, and $D^*$ over $t^*$. Nondimensional plots are reported to better visualize different evolution patterns.

At low relative humidity (the green-shaded region in Fig. 1b), change in substrate temperature does not alter the qualitative evolution of droplet. In this regime, the contact angle stays constant for most of droplet lifetime followed by a slight increase and a sharp decrease towards the end (Fig. 1b, bottom left). The modest rise in contact angle is attributed to the interplay of high evaporation rate of methanol and receding speed at the triple line^31^. Diameter and volume monotonically decrease during droplet lifespan.

Due to the high hygroscopic nature of methanol, at higher relative humidity, water vapor transfers into the droplet at the liquid–gas interface. Water adsorbing–absorbing and possibly condensing on the interface is reported in previous studies^30,33–37^. The growth in the concentration of water content changes the interfacial tensions and results in higher contact angle^34,36,37^. Unlike low relative humidity, substrate temperature plays a determining role in the regime of droplet evolution at high relative humidity of the surrounding. In the transition regime (red-shaded region), contact angle rises to a maximum value before gradually decreasing towards the end of droplet lifetime. Increasing contact angle demonstrates water uptake into droplet while methanol is evaporating. At the point of maximum contact angle, most of methanol has already evaporated and droplet consists mainly of water. However, some studies revealed that a small amount of residual methanol remains until the end of droplet lifetime^31,32^. Even though diameter and volume decrease monotonically, two obvious slopes are observed in their evolutions (Fig. 1b, top right). The two slopes correspond to two stages: the initial stage when merely methanol evaporates and the second stage when water mainly evaporates at a slower rate.

When the humidity of the environment is high and the substrate temperature is sufficiently low, another regime is observed. In condensation-dominated (blue-shaded) regime, contact angle monotonically increases until it reaches a plateau. Both diameter and volume converge to a non-zero value. Lower substrate temperature enhances water uptake through condensation by dropping the liquid-gas interface temperature below that of dew point^37^. In this regime, droplet comes to a quasi-steady state with a remaining droplet consisting mainly of water^31,32,34,36^.

### Regime classification

**Figure 2:**
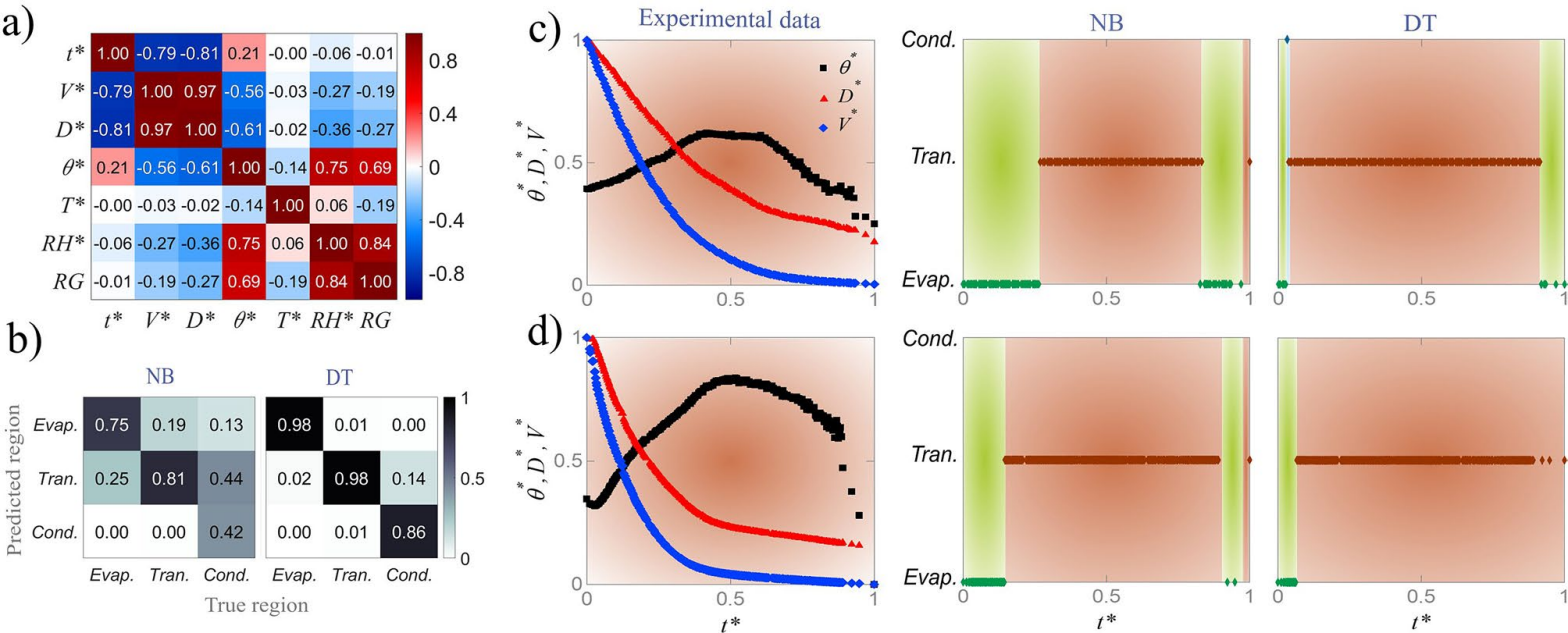
Results of regime classification: (**a**) correlation matrix for parameters in droplet evaporation; (**b**) results of test set for regime detection illustrated with confusion matrices for Naïve Bayes (NB) and decision tree (DT) algorithms; (**c**) point-by-point validation results with NB and DT classifiers for regime detection with experimental data of droplet evaporation for RH = 80% and T = 35 $^{\circ }$C; (**d**) point-by-point results of estimation set with NB and DT classifiers for regime detection with experimental data of droplet evaporation for experiment #4 (RH = 75% and T = 25 $^{\circ }$C) in estimation set. The colors in (**c**,**d**) correspond to the regime colors used in Fig. 1b.

We have used a classification algorithm to detect the regime of droplet evaporation as sketched in Fig. 1b. The classifier is trained with data on contact and diameter at each specific point in time and then classifies the regime of droplet evaporation. Dependence of variables is shown by the correlation matrix in Fig. 2a where *RG* stands for the regime of droplet evaporation. Diameter and volume are coupled for a spherical cap sessile droplet through the relation $V = (\pi /3)(D/2)^3(2+\cos \theta )(1-\cos \theta )^2$ which assumes slow quasi–static evaporation. $t^*$, $D^*$, and $\theta ^*$ are used as input variables and *RG* is the target variable. It is observed that the contact angle is highly proportional to humidity because the higher the humidity, the higher the amount of water uptake into drop. Higher water content increases the interfacial tension at the triple line which results in higher contact angles.

**Table 1:** Regime classification results on test set: standard metrics for two different classifiers.

Classifier	Accuracy	RG	Precision	Recall	F-score
NB	0.75	Evap.	0.74	0.65	0.6
		Trans.	0.81	0.78	0.79
		Cond.	0.46	0.93	0.61
DT	0.96	Evap.	0.99	0.97	0.97
		Trans.	0.97	0.96	0.96
		Cond.	0.83	0.89	0.85

The framework for detection of the regime of droplet evaporation characterizes the behavioral pattern of droplet by learning the values of contact angle and diameter at each specific point in time and classifying them to each regime. The model then labels the test and validation sets based on similar evolution observed previously during training. The ratio of training to test set is 80–20%. Two classifiers of Naïve Bayes (NB) and decision tree (DT) are trained and confusion matrices are used to compare the performance of classifiers on the test set (Fig. 2b). Precision, recall, F-score, and overall accuracy values (shown in Table 1) provide a comprehensive evaluation of the performance of each classifier on the test set. Based on the results shown in the Fig. 2b and Table 1, DT outperforms NB for all regimes of the test set. It is also observed that the detection of the condensation-dominated regime is challenging for both classifiers. Detection of evaporation-dominated regime reaches an F score value of 0.97 with decision tree classifier. For NB, around half (43%) of the points in the condensation-dominated regime are classified as transition regime (see Fig. 2b). Replacing diameter with volume slightly improves the results for both classifiers (6% on average) for this regime. This is due to a more discernible evolution of $V^*$ compared to $D^*$ towards the end of droplet lifetime for this regime. However, since measuring diameter is a more direct approach and also more convenient for the user, the model is trained with diameter.

The capability of the model to detect the regime of droplet evaporation under each specific condition (*RH* and *T*) is evaluated through a validation set. The validation step is performed on a single experiment at each time that is held out during training/testing. Validation results, averaged for each condition, are presented in Table 2. The accuracy and recall are the same for validation because at each condition there is only one true regime. Figure 2c illustrates a sample of validation for $RH = 80\%$ and $T = 35\,^{\circ }$C. The classifier assigns a region for each point in time based on the value of contact angle and diameter. The true regime for this condition is the transition regime (red). The red regions on the two plots on the right side of Fig. 2c represents the regions that are classified correctly as transition regime and the green regions show the regions that are incorrectly classified as the evaporation-dominated regime. It is observed that both classifiers correctly detect the region of the majority of the data points although DT demonstrates better performance. NB struggles at the beginning and end of droplet lifetime. This issue is less pronounced for DT.

**Table 2:** Regime classification results on validation set under each specific *RH* and *T* condition with two different classifiers.

Classifier	$RH(\%)$	15 $^{\circ }C$	23 $^{\circ }C$	35 $^{\circ }C$
NB	20	0.72 (± 0.04)	0.73 (± 0.08)	0.78 (± 0.06)
	50	0.80 (± 0.06)	0.62 (± 0.11)	0.63 (± 0.02)
	80	0.41 (± 0.10)	0.88 (± 0.06)	0.83 (± 0.08)
DT	20	0.98 (± 0.03)	0.96 (± 0.08)	0.96 (± 0.08)
	50	0.94 (± 0.10)	0.94 (± 0.03)	0.98 (± 0.03)
	80	0.74 (± 0.23)	0.97 (± 0.05)	0.94 (± 0.07)

**Table 3:** Results of regime estimation for four experiments under new conditions unseen by the model.

Experiment	$RH(\%)$	$T(^{\circ }C)$	NB		DT	
			Evap.	Trans.	Evap.	Trans.
1 (X)	30	15	0.54	0.46	0.67	0.33
2 (X)	33	23	0.61	0.39	0.79	0.21
3 (X)	65	35	0.32	0.68	0.16	0.84
4 (*)	75	25	0.18	0.82	0.07	0.93

The true regime of each experiment is shown on the regime map in Fig. 1b.

The capability of the model to estimate the regime of droplet is evaluated on an estimation data set from conditions that are unseen by the model and do not contribute to the model training, testing, and validation. The values of *RH* and *T* for these conditions are randomly selected in the range of 20%$<RH<$80% and 15 $^{\circ }$C $<T<35\,^{\circ }$C (shown with cross and star marks on the regime map of Fig. 1b). The model classifies each experiment under each regime with different ratios as shown in Table 3. For example, Experiment 1 with *RH* of 30% and *T* of 15 $^{\circ }$C is close to the boundary of evaporation-dominated and transition regimes. With NB, 54% of the data points in this experiment are classified as evaporation-dominated regime and 46% as transition regime. This is expected due to the location of Experiment 1 on the regime map. It should be noted that the lines on the regime map are approximate boundaries. Figure 2d demonstrates regime estimation of all data points of experiment 4 with both classifiers. The shown evolution of contact angle is not similar to any of the evolutions shown in Fig. 1b subfigures. In fact, contact angle decreases at the beginning and then starts rising. Experiment #4 falls in transition regime. That is the reason that the profile for experimental data in Fig. 2d is colored in red for the true regime of experiment #4. As it is seen, NB and DT correctly classify 84 and 93% of the data points in experiment 4 to transition regime. The green regions represent the data points that are incorrectly classified as evaporation-dominated regime.

### Relative humidity estimation

**Figure 3:**
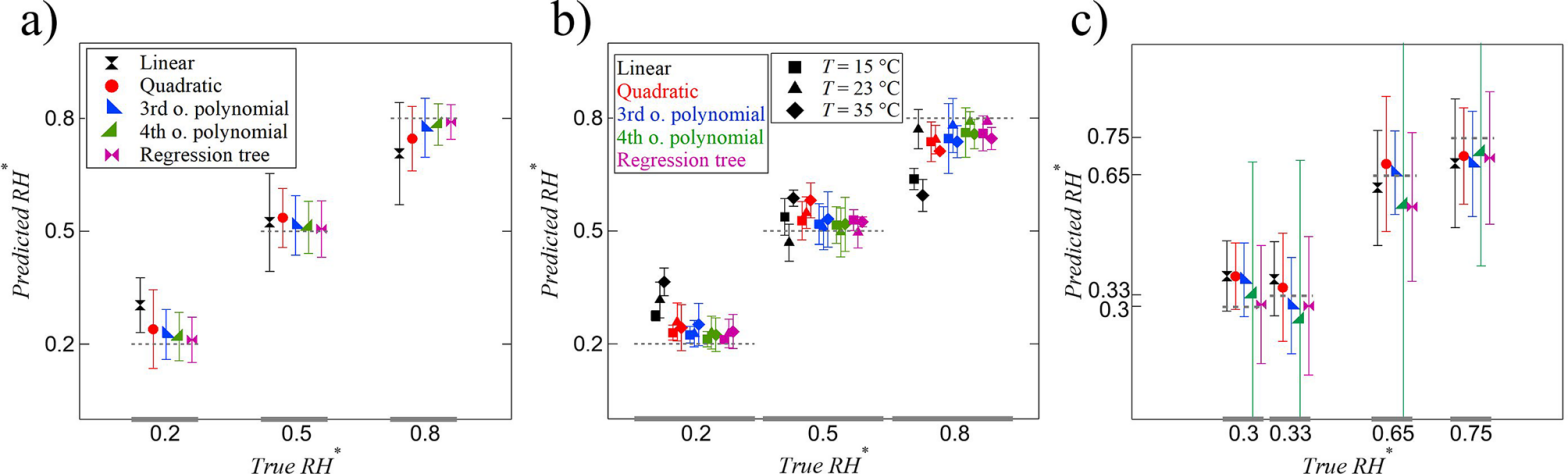
Relative humidity regression results: (**a**) test set; (**b**) validation; (**c**) estimation. Markers for all colors in (**b**) represent different temperatures as shown in legend. Markers and colors in (**c**) are the same as in (**a**).

In this section, we show the ability of the model to detect environmental humidity by analyzing the evolution of contact angle and diameter through regression algorithms. Polynomial regression with four different orders (linear, quadratic, third-order, and fourth-order) and regression tree are used for training. The coefficient of determination ($R^2$) increases from 0.66 for linear up to 0.93 for fourth-order polynomial regression. The test results for all five regression methods are shown in Fig. 3a. The horizontal axis shows the true value of $RH^*$ and the vertical axis shows the estimated values averaged over all the points for each $RH^*$. As it can be seen, the higher the order of polynomial regression, the closer the average estimation to the ground-truth value and the smaller the error bar. Furthermore, regression tree performs more accurately compared to all polynomial regression methods. Model performance under each specific condition through validation set is shown in Fig. 2b. The consistent colors throughout Fig. 3a–c represent different regression methods and different markers are used for each substrate temperature in Fig. 3b. Based on the results shown in Fig. 3b, all methods except linear regression produce reasonably accurate results. The validation results get closer to actual values as the order of the polynomial regression increases. Nonetheless, it must be noted that higher order polynomial regression increases the computational cost as well as the chance of over-fitting. Performance of regression tree is comparable to third-order and fourth-order polynomial. The capability of different regression methods to estimate new humidity values by analyzing the time evolution of contact angle and diameter is presented in Fig. 3c. The new relative humidity values (30, 33, 65, and 75%) are randomly selected in the range of 20–80%. It is noteworthy that the model has not seen any data of droplet evolution under these *RH* values during training, testing, or validation. It is seen that, unlike testing and validation where increasing the order of polynomial or complexity of the model (i.e., regression tree) produces more accurate results, higher order polynomials do not result in better estimation of unseen conditions. As a matter of fact, linear, quadratic, and third-order polynomials estimate more accurately. This is a common issue when the model fits the training data very well and it negatively affects the model performance on the new data set. Figure 3c clearly indicates over-fitting with fourth-order polynomial.

### Diameter and contact angle estimation

**Figure 4:**
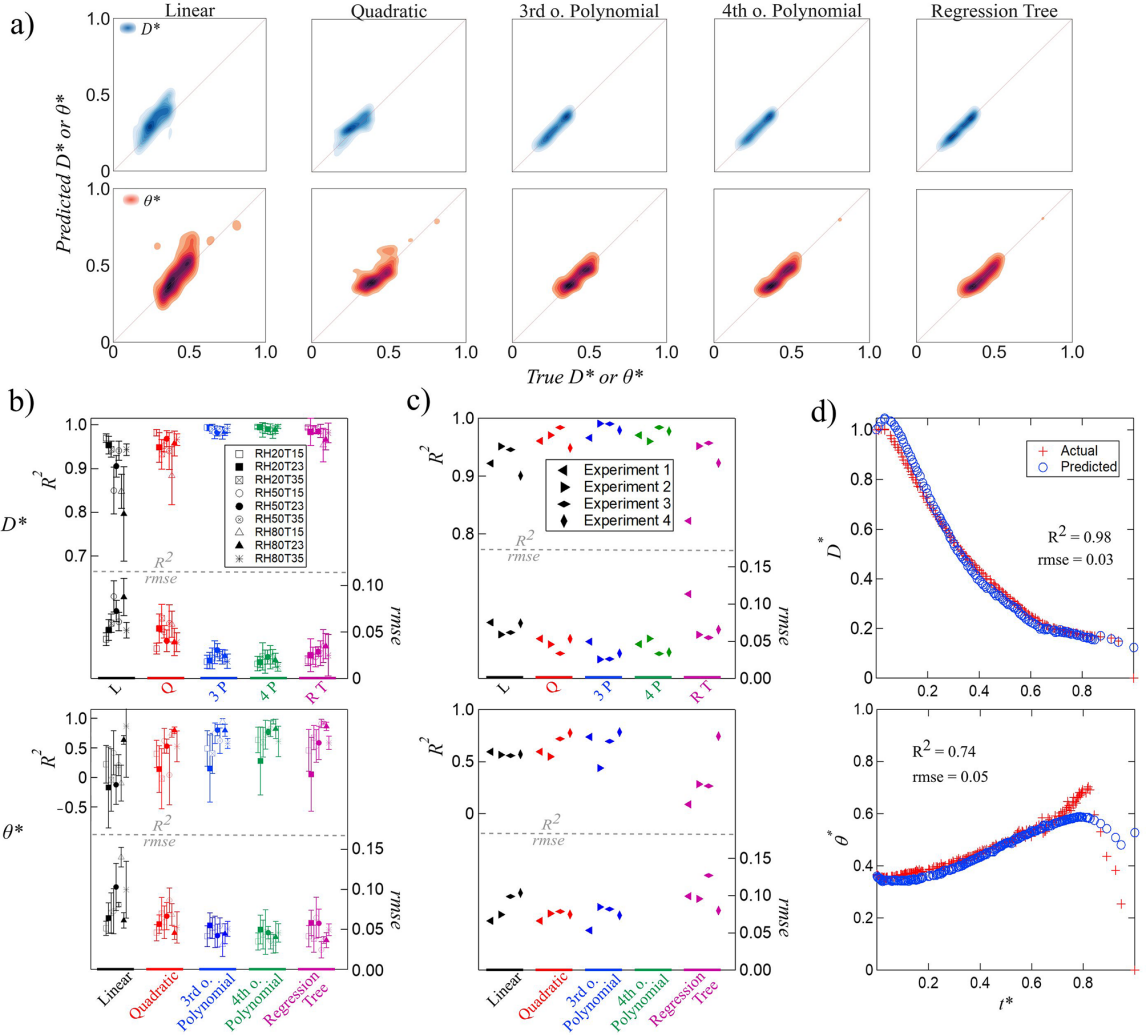
Diameter (D*) and contact angle $(\theta ^*)$ regression results: (**a**) test set; (**b**) validation set; (**c**) estimation set; (**d**) diameter estimation with quadratic regression for E3 (top) and contact angle estimation with third-order polynomial regression for E1 (bottom).

In this section, the capability of the model to estimate the continuous evolution of contact angle and diameter over time is evaluated through regression algorithms. This means that the model estimates the evolution of diameter and contact angle at each time increment (approximately one second apart). The input variables include $T^*$, $RH^*$, $t^*$, and $\theta ^*$ (or $D^*$) and the target variable is $D^*$ (or $\theta ^*$). By increasing the order of the polynomial, the coefficient of determination, $R^2$, for training improves from 0.87 to 0.99, and from 0.78 to 0.96 for diameter and contact angle estimation, respectively. The performance of five different regression methods on the test set is presented in Fig. 4a. The first row represents the results when $D^*$ is the target variable and the second row illustrates the results for $\theta ^*$ as the target variable. The closer the distribution of data to the diagonal line in these plots, the better the performance of the model on the test set. Based on the results shown in Fig. 4a, the diameter test results saturate after third order polynomial while for contact angle the performance keeps improving when increasing the degree of polynomial from third to fourth.

The validation results are summarized in Fig. 4b for $D^*$ (top) and $\theta ^*$ (bottom) in terms of $R^2$ and root mean square error (*rmse*). With $D^*$ being the target variable, an average $R^2$ of 0.8 or higher and average *rmse* less than 0.1 are achieved for all nine conditions. Going from linear to quadratic to third order polynomial increases and decreases the value of $R^2$ and *rmse*, respectively. The profiles of $R^2$ and *rmse* exhibit saturation, and further increase in the order of the polynomial does not improve model performance on validation data. This is consistent with the test results where model performance saturates at third order. Furthermore, regression tree demonstrates accuracy comparable to third-order and fourth-order polynomials. By comparing the range of axes in Fig. 4b-top with bottom, it is obvious that $R^2$ values are generally lower (hardly reaching 0.7) and error is higher when $\theta ^*$ is the target variable. In fact, there are a few instances where the average $R^2$ turns negative, suggesting that the overall estimation of the model is worse than an estimation with a constant average value.

The performance of the model on estimating the evolution of $\theta ^*$ and $D^*$ versus time under four new conditions that did not contribute to model training, testing, or validation is shown in Fig. 4c. One value of $R^2$ and *rmse* are reported for each condition (or experiment) which shows the overall quality of the fit. Higher coefficients of determination and lower *rmse* values demonstrate the better performance of the model in estimating $D^*$ then $\theta ^*$ evolution. Based on the results shown in Fig. 4c, third order polynomial regression has the best performance in estimating diameter. The results become less accurate with fourth-order polynomial which suggests over-fitting. It is interesting to note that regression tree, which had higher accuracy during testing and validation, is outperformed even by linear regression during estimation. The evolution of diameter versus time estimated by quadratic regression for Experiment 3 is depicted in Fig. 4d. As it can be seen, even with a quadratic regression, the model estimates the evolution of $D^*$ quite accurately for an unseen condition. Considering the range of values on axes of Fig. 4c-top and bottom, estimating the evolution of contact angle is more challenging for the model. Unlike estimating diameter, increasing the order of polynomials has a negligible effect. The accuracy of the model stays almost constant for linear, quadratic, and third order. However, it worsens drastically for fourth-order polynomial due to over-fitting the data. The $R^2$ and *rmse* values for contact angle estimation with fourth-order polynomial fall outside the range shown in the plot. Since the estimation of $\theta ^*$ is generally more challenging for the model, the effect of over-fitting is more noticeable compared to $D^*$ estimation. The overall better performance of the model for diameter estimation compared to contact angle estimation is due to the fact that diameter evolution is relatively smooth and therefore easier to estimate whereas contact angle evolution changes substantially under different conditions. Figure 4d-bottom illustrates contact angle evolution over time estimated with third-order polynomial for Experiment 1 which is in the evaporation-dominated regime. Although the maximum value of contact angle is underestimated with 3rd order polynomial, the time corresponding to the maximum contact angle is estimated accurately. Also, there is a good agreement between the estimated and actual contact angle evolution during most of droplet lifetime with $R^2$ value of 0.74 and *rmse* of 0.05.

## Discussion

In this study, we have analyzed the complex physics of sessile droplet evaporation (which starts with a single component and turns into a binary system due to the transfer of a second component i.e., water) through machine learning, classification and regression algorithms. Four different parameters pertaining to droplet evaporation, namely: regime of droplet evaporation, level of surrounding humidity, time evolution of droplet base diameter, and time evolution of droplet contact angle, are estimated. Point-by-point analysis of droplet profile enables time-dependent estimations given a limited number of data points. This means that the model does not need the entire evolution profile of a droplet to make an estimation. Instead, only a few (or even a single) data points are (is) sufficient for estimation, although more data points result in more accurate estimation. The model estimation capability is then assessed on the data that do not contribute to training, testing, or validation.

Two different classifiers are utilized to estimate the regime of droplet evaporation. NB is chosen as a simple easy-to-interpret algorithm, while DT serves as a more powerful algorithm. As expected, DT outperforms NB due to a more robust internal structure at the expense of computational cost and transparency. Both classifiers showed impressive performance (minimum 75% accuracy) on estimating the regime of droplet. Knowledge on droplet evaporation regime is necessary for compatible designs in numerous industries such as droplet-based biosensors or ink-jet printing.

Additionally, level of surrounding humidity, time evolution of droplet base diameter, and time evolution of droplet contact angle are estimated through regression techniques. Polynomial regressors, as well as regression tree, are trained through point-by-point analysis of droplet evolution. The model performance improved by increasing the order of the polynomial and using regression tree for training, test, and validation sets. However, when estimating the new conditions unseen by the model, fourth-order polynomial and regression tree suffered from data over-fitting. The best performance of the model is achieved by third-order polynomial. In general, the model estimation results are more accurate when estimating diameter evolution compared to contact angle estimation. This is due to smoother, hence easier to estimate, evolution of diameter with time. The sharp changes in $\theta ^*$ under different conditions make the estimation of its evolution challenging for the model. Information of this type is of great importance for technologies such as ink-jet printing, or droplet-based biodiagnostics where the estimations can provide critical information on the base diameter or contact angle of the droplet at a specific time.

In the current work, behavior of a sessile droplet transitioning from a single fluid into a binary mixture following transfer of a second component from the atmosphere is studied through data-driven techniques. The model demonstrated promising performance detecting the regime of droplet evolution, the humidity level surrounding droplet, and time evolution of diameter and contact angle. The current case study demonstrates the capability of the proposed model to analyze complex interfacial fluid mechanics problems through machine learning algorithms. Although we have estimated four parameters of droplet evaporation by this model, these kinds of techniques can be expanded to perform a wide range of estimations. Our preliminary study opens up new ways to study binary or multi-component droplet evolution which might lead to better analyzing the complex physics of the problem.

## Methods

### Experimental setup and procedures

The experiments are carried out in a chamber (with dimensions $127\times 127\times 76$ mm$^3$) of drop shape analyzer (DSA 100) from KR$\ddot{\mathrm{U}}$SS. The relative humidity inside the chamber is controlled and the temperature is kept at room temperature (i.e., 23 $^{\circ }\mathrm{C}$). The bottom side of the chamber is equipped with a Peltier plate (electrical system and a temperature bath) to control the substrate temperature. The top of the chamber has a small hole for passing the syringe. Both sides of the chamber have transparent windows for visualization purposes. An LED light is used for illumination and a CCD camera is utilized to capture the time evolution of droplet profile (Fig. 1a). The whole setup is mounted on an anti-vibration table to eliminate environmental disturbances.

Methanol is purchased from Fisher Scientific with a purity of 99.8%. The glass substrates are coated with a very thin PDMS (polydimethylsiloxane) layer to achieve spherical and reproducible droplets with measurable contact angles. In order to ensure that methanol does not interact with the PDMS coating, multiple methanol droplets are successively deposited at the same location on the substrate and let evaporate. No change is observed in the initial contact angle of droplets or in their evolution during evaporation. The relative humidity inside the chamber and the temperature of the substrate are set to desired values and enough time is passed to make sure quasi-steady state is achieved. Three values of relative humidity: 20%, 50%, and 80% alongside three values of substrate temperature: 15 $^{\circ }\mathrm{C}$, 23 $^{\circ }\mathrm{C}$ (room temperature), and 35 $^{\circ }\mathrm{C}$ are tested. A drop of methanol is gently deposited on the glass substrate. Droplet volume is under 5 $\upmu$l in order to keep droplet size below capillary length. The corresponding volume to the capillary length for methanol droplet is around 45 $\upmu$l. The evaporation process of methanol droplet is recorded by the CCD camera at 50 frames per second. All experiments under each relative humidity and substrate temperature condition are repeated five to ten times to ensure the reproducibility of the data. A KR$\ddot{\mathrm{U}}$SS Drop Shape Analyzer software is utilized to measure the time evolution of contact angle ($\theta$), base diameter (*D*), and volume (*V*). Due to the observed differences between the right and left contact angle values, elliptical fit is used for contact angle and volume measurements. For better analysis of droplet behavior, all dimensional parameters are nondimensionalized as followed: $D^*=D/D_0$, $V^*=V/V_0$, $t^*=t/t_f$; where $D_0$, $V_0$, and $t_f$ are initial diameter, initial volume, and total evaporation time, respectively.

### Data acquisition

The data for the model is generated by experiments of methanol droplet evaporation under various environmental conditions. Nine different conditions are created by a combination of three levels of surrounding relative humidity: 20%, 50%, and 80% with three substrate temperatures: 15 $^{\circ }$C, 23 $^{\circ }$C, and 35 $^{\circ }$C. For each of these nine conditions, the relative humidity in the chamber and substrate temperature is set to the desired values and enough time is passed to ensure quasi-steady state. Then a droplet of methanol is gently deposited on the substrate and the evolution of droplet base diameter, contact angle, and volume is recorded over time with a CCD camera of Drop Shape Analyzer (see Fig. 1a inset). Each point in time with corresponding contact angle, diameter, and volume is considered a data point for the model.

### Data partitioning and processing

The data generated under nine different conditions mentioned in the previous section are used to train, test, and validate the model. The droplet evaporation experiments for each of these nine conditions are repeated five to ten times to make sure that the results are consistent and reproducible. We carried out 60 droplet evaporation experiments under these nine conditions which created a total number of 10,850 data points that are used for training, testing and validating the model. There are additional 761 data points that are used to assess the performance of the model at the final step. The environmental conditions under which these data points are generated are completely different than the other 10,850 data points used for training, testing, and validating the model.

### Training and testing

The data for training, testing, and validation are generated by methanol droplet evaporation under nine different conditions which are the combinations of three levels of surrounding humidity: 20%, 50%, and 80% with three substrate temperatures: 15 $^{\circ }$C, 23 $^{\circ }$C, and 35 $^{\circ }$C. The behavior of droplet under the same condition is similar between experiments however it is not exactly the same due to microscopic defects of the surface, initial conditions, etc. The data from a single experiment is held out at the beginning of training as a validation set. The remaining data from 59 experiments is partitioned into a training set and a test set with 80–20% ratio. It should be noted that multiple training to test set ratios are examined to ensure the convergence of the model which is discussed in the “Performance criteria” section.

### Cross validation

A 10-fold cross-validation is carried out. The results for the test set show one of 10 scenarios from the test set. Cross-validation is performed to ensure all the results for training and test set are similar to each other and there is no anomaly in the data.

### Validation

Once the model is trained and tested, it is then examined on the validation set that was held out at the beginning of training. The validation set that is kept out, is the data pertaining to one whole experiment out of 60 experiments. This means that the model has not seen the data for the specific experiment in the validation set. Validation is performed to specifically evaluate the performance of the model under each specific condition of surrounding humidity and substrate temperature. This procedure is repeated 60 times until each experiment is held out once and validated. Validation results are averaged over all experiments under each specific *RH* and *T* condition.

### State estimation

There are additional 761 data points, called estimation set, that are generated by four experiments of methanol droplet evaporation. It must be noted that the machine has not seen any data of droplet evaporation under these new conditions. The data in the estimation set does not contribute to the framework during training, testing, or validation. The surrounding humidity and substrate temperature for these new conditions are randomly selected to be in the range within which the model is trained (i.e., 20% $<RH<$ 80% and 15 $^{\circ }$C$<T<35^{\circ }$ C).

### Performance criteria

The performance criteria of the model are reported by standard metrics. For classification, the confusion matrix of the model is used as well as the precision, recall, F score, and overall accuracy values for each regime. For validation, the accuracy values are the same as recall values for each combination of *RH* and *T* because there is only one true regime for each validation set. For regression methods, coefficient of determination ($R^2$) is reported to show how well the model fits the data in the training set. For *RH* testing, validation, and estimation, the actual *RH* of the environment is compared against the estimated value of *RH*. When $D^*$ (or $\theta ^*$) is the target variable, the test results are demonstrated as estimated values versus actual values. The validation and estimation results are reported by $R^2$ that shows the quality of the fit; the proportion of variance in the target variable which is predictable from the input variables; and root mean squared error (*rmse*) which represents how much the estimation is off on average when estimating the average target variable. We have also tested the model convergence for both classification and regression algorithms. We trained the model by considering only fractions of the available data i.e., 80%, 70%, 60%, and 50%, and observed that the results for the test set remain unchanged. This proves the fact that the number of data points used for machine learning of this specific problem is converged.

### Classifiers

We have used Naïve Bayes Classifier^64–67^ as a simple and easy-to-interpret algorithm. Since the algorithm is simple, there is less chance for over-fitting the data, it is faster and needs a smaller memory footprint. However, the restrictive underlying assumptions compromise its accuracy for real case scenarios when the variables are not fully independent of each other.

Bagged Decision Tree^68–70^ with 250 trees is also used. It is a powerful classifier with built-in support for cross-validation and a specialized function to measure feature importance. However, it results in complex models that are not very transparent. It is often hard to understand how it makes estimations.
